# Improved Pulse-Controlled Conductance Adjustment in Trilayer Resistors by Suppressing Current Overshoot

**DOI:** 10.3390/nano10122462

**Published:** 2020-12-09

**Authors:** Hojeong Ryu, Sungjun Kim

**Affiliations:** Division of Electronics and Electrical Engineering, Dongguk University, Seoul 04620, Korea; hojeong.ryu95@gmail.com

**Keywords:** resistive switching, X-ray photoelectron spectroscopy, synaptic device, metal oxide, current overshoot

## Abstract

In this work, we demonstrate the enhanced synaptic behaviors in trilayer dielectrics (HfO_2_/Si_3_N_4_/SiO_2_) on highly doped n-type silicon substrate. First, the three dielectric layers were subjected to material and chemical analyses and thoroughly investigated via transmission electron microscopy and X-ray photoelectron spectroscopy. The resistive switching and synaptic behaviors were improved by inserting a Si_3_N_4_ layer between the HfO_2_ and SiO_2_ layers. The electric field within SiO_2_ was mitigated, thus reducing the current overshoot in the trilayer device. The reset current was considerably reduced in the trilayer device compared to the bilayer device without a Si_3_N_4_ layer. Moreover, the nonlinear characteristics in the low-resistance state are helpful for implementing high-density memory. The higher array size in the trilayer device was verified by cross-point array simulation. Finally, the multiple conductance adjustment was demonstrated in the trilayer device by controlling the gradual set and reset switching behavior.

## 1. Introduction

Resistive switching memory is very attractive for a wide range of applications due to its various resistive switching characteristics stemming from a number of resistive switching materials by easily tunable resistive switching parameters such as on-resistance, off-resistance, and operation voltage [1,2,3,4,5]. Moreover, its simple structure, such as metal–insulator–metal (MIM) with 4F^2^ (F is feature size), can be scaled down via a lithography process [1,2,3,4,5,6]. Further, the multiple resistance states triggered by electrical pulses can be used for high-density memory. Finally, the good retention properties, such as NAND flash and high endurance, provide an edge over other competing memory products. The types of resistive switching should be characterized depending on the possible applications, such as in storage class memory, neuromorphic devices, and logic devices. Among them, a neuromorphic device using resistive switching memory is attracting considerable attention [7,8,9,10]. To meet the needs of efficient data processing in the era of big data, neuromorphic computing provides a major breakthrough that can replace the existing Von Neumann computing. In particular, neuromorphic systems are specialized in data processing, such as complex pattern recognition. Moreover, they take an energy-efficient approach by carrying out data processing in a parallel manner. In a neuromorphic system, the conductance of the resistive switching memory cell placed on a cross-point array has multiple states and can be updated and controlled by the input pulse from the neuron circuit. The conductance control of resistive switching is similar to the synaptic weight adjustment in biological synapses in the human nervous system.

Resistive switching and artificial synaptic behaviors are observed in many insulators, such as oxide [11,12,13], nitride [13,14,15], organic materials [16], and 2D materials [17]. Among them, metal–oxide-based resistive switching memories such as HfO_2_ have proven to have the best resistive switching performances in terms of, e.g., endurance, retention, and variability. Excellent resistive switching has been reported when using metal bottom electrodes [18,19]. HfO_2_-based resistive switching memory with ITO electrode can also be used for flexible and transparent electronic devices [20]. On the other hand, the HfO_2_-based resistive memory with a silicon bottom electrode has not yet been reported as superior to the metal bottom electrode. However, the metal–oxide–semiconductor structure has other advantages, such as self-rectification and low-power operation. The most effective way to enhance resistive switching is to design multiple dielectric stacks [21]. The use of an oxygen reservoir, such as a TiO*_x_* layer, is popular in metal–oxide-based resistive switching memory. Abundant oxygen vacancies are created in the main resistor for resistive switching. A tunnel barrier, such as SiO_2_ and Al_2_O_3_, with a large band gap, can enhance the resistive switching properties by reducing the operation current and increasing the nonlinearity of the I–V curve in the low-resistance state (LRS) [22,23]. The SiO_2_ layer can be easily formed when using silicon substrate as the bottom electrode and different methods such as native oxide, thermal oxide, and chemical vapor deposition (CVD). Another advantage of inserting the tunnel barrier with a high band gap is a reduction in the LRS current [21,22]. To employ the advantageous tunnel barrier in resistive switching, the insulating property of the SiO_2_ layer is maintained after the forming and set processes. If the excess electric field is applied on the tunnel barrier with a high compliance current, breakdown of the tunnel barrier can occur. Therefore, the use of a careful device stack design is necessary to ensure a stable tunnel barrier layer; for example, the thicknesses of the tunnel barrier and the main resistor are important. In addition, the dielectric constant should be considered to properly distribute the electric field throughout multiple dielectric layers.

In this work, we fabricated a trilayer (HfO_2_/Si_3_N_4_/SiO_2_) resistive switching memory device and demonstrated low current switching by suppressing the current overshoot and nonlinear I–V curves in the LRS. The trilayered dielectric stacks were confirmed via high-resolution transmission electron microscopy (TEM) and X-ray photoelectron spectroscopy (XPS) before electrical characterization. Through a comparative study with a control group without a Si_3_N_4_ layer, it was verified that the Si_3_N_4_ layer can relieve the concentration of the electric field in SiO_2_. Finally, we demonstrated the improved synaptic behaviors by achieving gradual conductance control in the trilayer structure compared to the device without a Si_3_N_4_ layer.

## 2. Materials and Methods

The Ni/HfO_2_/Si_3_N_4_/SiO_2_/Si device was prepared as follows: The ion implantation was conducted in the Si substrate to increase the conductivity on the single crystalline Si surface as the bottom electrode. Phosphorus (P) as an impurity was used to form an n-type Si bottom electrode, where the dose and energy were 5 × 10^15^ cm^−2^ and 40 keV, respectively. The Si lattice damage caused by ion implantation was cured by the annealing process. Next, a 2.5-nm-thick SiO_2_ film was deposited via low-pressure chemical vapor deposition (LPCVD) by reacting SiH_2_Cl_2_ (40 sccm) and N_2_O (160 sccm) at 785 °C after removing native oxide through HF cleaning. Then, a 3.5-nm-thick Si_3_N_4_ layer was deposited via LPCVD by reacting SiH_2_Cl_2_ (30 sccm) and NH_3_ (100 sccm) at 785 °C. After that, a 3.5-nm-thick HfO_2_ layer was deposited by atomic layer deposition (ALD) system by reacting tetrakis (ethylmethylamino) hafnium (TEMAH) and ozone (O_3_) at 300 °C. Finally, a 100-nm-thick Ni top electrode was deposited by a thermal evaporator and patterned by a shadow mask containing circular patterns with a diameter of 100 μm. A Ni/HfO_2_/SiO_2_/Si device was prepared as a control device in the same way, except for the Si_3_N_4_ layer.

The electrical properties were characterized both in DC mode using a Keithley 4200-SCS semiconductor parameter analyzer (Keithley Instrumnets, Cleveland, OH, USA) and in pulse mode using a 4225-PMU ultrafast module (Keithley Instrumnets, Cleveland, OH, USA) During the measurements, a bias voltage and pulse were applied to the Ni top electrode, while the Si bottom electrode was grounded. XPS depth analysis was conducted with a Nexsa (ThermoFisher Scientific, Waltham, MA, USA) with a Microfocus monochromatic X-ray source (Al-Kα (1486.6 eV)), a sputter source (Ar+), an ion energy of 1 kV, and a beam size of 100 µm × 100 µm.

## 3. Results and Discussion

**Figure 1:**
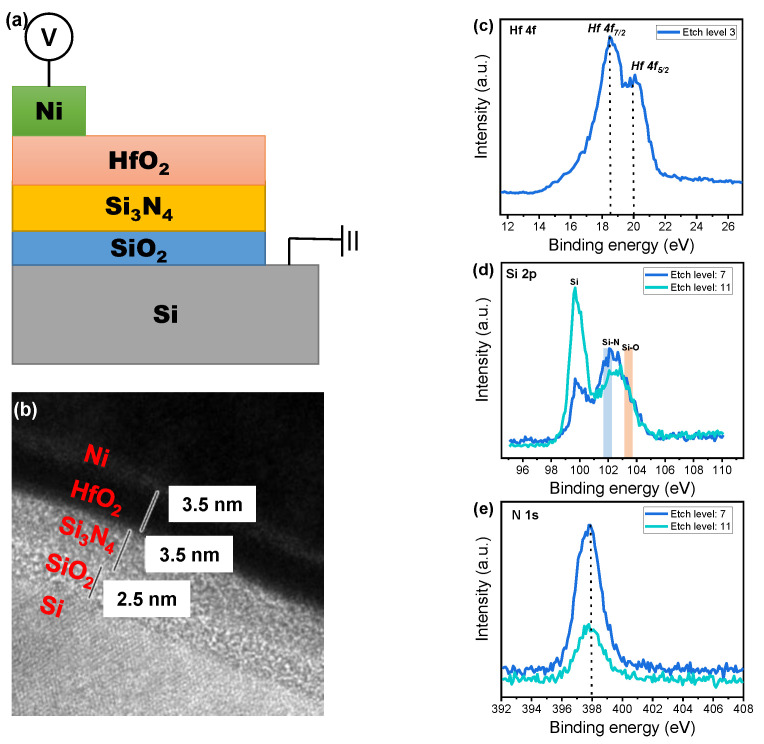
Device configuration and material analysis of the Ni/HfO_2_/Si_3_N_4_/SiO_2_/Si device. (**a**) Schematic drawing of the device stack; (**b**) TEM image; (**c**) XPS Hf 4f spectra at etch level 3; (**d**) XPS Si 2p spectra at etch levels 7 and 11; and (**e**) XPS N 1s spectra at etch levels 7 and 11.

Figure 1a,b respectively shows the schematics and a TEM image of the Ni/HfO_2_/Si_3_N_4_/SiO_2_/Si device. Single crystalline Si substrate and amorphous HfO_2_, Si_3_N_4_, and SiO_2_ layers could be observed in the TEM image. In addition, the TEM image provides information about the exact film thicknesses of HfO_2_ (3.5 nm), Si_3_N_4_ (3.5 nm), and SiO_2_ (2.5 nm). The energy-dispersive X-ray spectra (EDS) line scan was obtained through scanning transmission electron microscopy (STEM) and is shown in Figure S1. Next, the XPS depth profile of HfO_2_/Si_3_N_4_/SiO_2_/Si was investigated to determine the elements in each layer. Figure 1c shows the XPS spectra Hf 4f of HfO_2_ as the first dielectric layer [24]; Hf 4f is typically composed of a 4f 5/2 and 4f 7/2 spin–orbit doublet, which are respectively centered at 20 and 18.5 eV. This result is consistent with existing literature about HfO_2_ on a Si substrate [24]. Figure 1d shows the Si 2p spectra for the Si_3_N_4_ layer, SiO_2_ layer, and Si substrate. The peak intensity that is located at about 99.5 eV is higher at the deeper etching level (level 11) than it is at level 7. This indicates that the Si substrate is more exposed by X-ray beams at the deeper etching level (level 11) [21]. Moreover, the peak point at etch level 11 is shifted to the right compared to that at etch level 7, indicating that the Si–O bond located at 103.5 eV is increased at level 11 [25]. Figure 1e shows the N 1s spectra at level 7 and level 11, where the peak is centered at about 398 eV [26]. The peak intensity at level 11 is much weaker than that at level 7. This result is consistent with the Si 2p result shown in Figure 1d.

**Figure 2:**
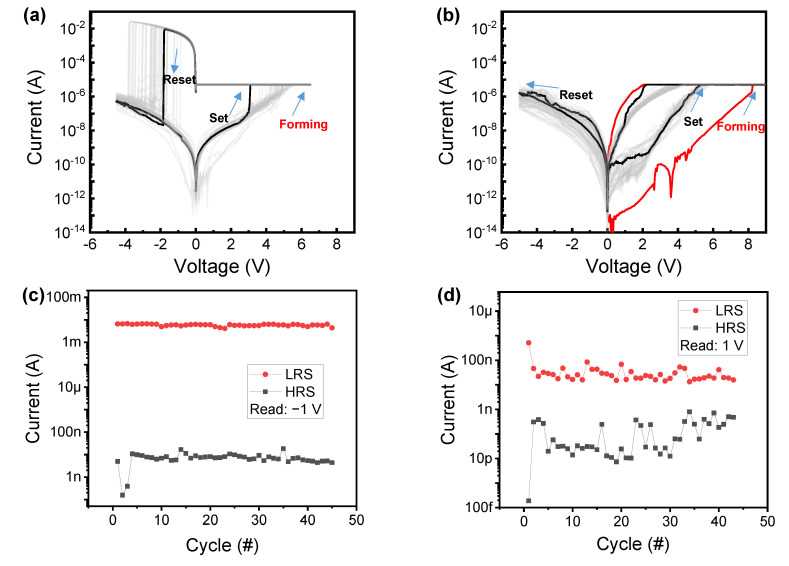
I–V characteristics of the (**a**) Ni/HfO_2_/SiO_2_/Si device and (**b**) Ni/HfO_2_/Si_3_N_4_/SiO_2_/Si device including forming, set, and reset processes; cycling data of I–V characteristics of (**c**) Ni/HfO_2_/SiO_2_/Si device and (**d**) Ni/HfO_2_/Si_3_N_4_/SiO_2_/Si device.

Figure 2a,b shows the I–V characteristics of the Ni/HfO_2_/SiO_2_/Si and Ni/HfO_2_/Si_3_N_4_/SiO_2_/Si devices. For a fair comparison, the compliance current (CC) of 5 μA is applied to both devices. The initial cells are activated with the positive bias DC sweep. The current is significantly increased during the reverse sweep, which indicates that soft breakdown occurs within the dielectrics. The CC can protect the device from permanent breakdown. Subsequently, the reset process is conducted by the negative bias sweep, causing the state of the device to be changed to the high-resistance state (HRS). This process can be explained by the rupture of the conducting path in dielectrics. Then, the set process follows to make the state of device be the LRS again. The HRS and LRS of the device can be repeatedly switched in the repetitive set and reset process. It should be noted that high current (~10 mA) flows within the Ni/HfO_2_/SiO_2_/Si device in the LRS. The current is very high despite the fact that 5 μA is applied on the Ni/HfO_2_/SiO_2_/Si device during the forward and reverse sweep in the LRS under the positive bias. Subsequently, the high LRS current without CC in a negative bias is the real current level. This suggests that current overshoot occurs during the set process, meaning that the LRS current cannot be tightly controlled by CC. An abrupt transition is observed during the reset process, indicating that the conducting path is ruptured at once. The I–V characteristics of the Ni/HfO_2_/Si_3_N_4_/SiO_2_/Si device are substantially different from those of the Ni/HfO_2_/SiO_2_/Si device, as shown in Figure 2b. The LRS current in a negative region is lower than the CC of 5 μA. This implies that the current overshoot is suppressed during the set process at a positive region. The bipolar resistive switching is driven by the temperature and electric field [27,28,29,30]. The Ni/HfO_2_/SiO_2_/Si device shows abrupt reset with high current, indicating that Joule heating is the dominant mechanism of the reset process. On the other hand, the electric field may be more important for the Ni/HfO_2_/Si_3_N_4_/SiO_2_/Si device considering the switching at low current.

Figure 2c,d shows the cycling trend of the Ni/HfO_2_/SiO_2_/Si and Ni/HfO_2_/Si_3_N_4_/SiO_2_/Si devices, respectively. Time series statistical analysis could provide the indirect information of filament evolution [31,32]. Both HRS and LRS are stable except for the initial few points during the cycling for the Ni/HfO_2_/SiO_2_/Si device. This indicates that the large size of conducting filament could be uniformly formed and ruptured. On the other hand, the read current of the Ni/HfO_2_/Si_3_N_4_/SiO_2_/Si device has larger variation during the cycling, and the read current in the HRS is increased. The larger variation is probably due to the fact that the filaments are formed and ruptured in multiple layers (HfO_2_/Si_3_N_4_/SiO_2_), and these formations and ruptures would be quite random processes spatially inside the insulators.

**Figure 3:**
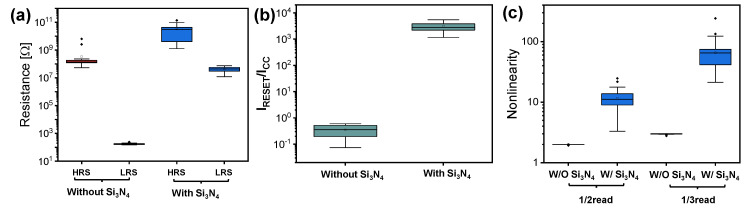
Statistical distributions of (**a**) high-resistance state (HRS) and low-resistance state (LRS) resistance, (**b**) I_RESET_/I_CC_, and (**c**) nonlinearity for Ni/HfO_2_/SiO_2_/Si and Ni/HfO_2_/Si_3_N_4_/SiO_2_/Si devices.

Figure 3a shows the statistical distribution of the Ni/HfO_2_/SiO_2_/Si and Ni/HfO_2_/Si_3_N_4_/SiO_2_/Si devices in the LRS and HRS. The LRS resistance of the Ni/HfO_2_/Si_3_N_4_/SiO_2_/Si device is much higher than that of the Ni/HfO_2_/SiO_2_/Si device. However, the variations of the LRS and HRS of the Ni/HfO_2_/Si_3_N_4_/SiO_2_/Si device are worsened. Figure 3b shows the ratio between reset current (I_RESET_) and CC. From this ratio, we can obtain information on how much CC suppresses the overshoot current during the set process. I_RESET_ is rather smaller than CC in the Ni/HfO_2_/Si_3_N_4_/SiO_2_/Si device. However, the I_RESET_/I_CC_ ratio of the Ni/HfO_2_/Si_3_N_4_/SiO_2_/Si device is more than 1000. Other advantages of the Ni/HfO_2_/Si_3_N_4_/SiO_2_/Si device are its high nonlinear I–V characteristic and its low-current operation. The nonlinearity is defined as the ratio between the current at read voltage (V_READ_) and the current at half read voltage (1/2·V_READ_) for the half bias scheme in the cross-point array (Figure S2). Further, the nonlinearity is defined as the ratio between the current at V_READ_ and the current at 1/3·V_READ_ for the 1/3 read scheme. The LRS resistance is the main leakage path in the cross-point array structure when reading the target cell with the HRS. Therefore, the current at 1/2·V_READ_ or the current at 1/3·V_READ_ should be suppressed to reduce crosstalk among the cells. Figure 3c shows the nonlinearity of both devices when applying the 1/2 read scheme and the 1/3 read scheme. The nonlinearities of the Ni/HfO_2_/SiO_2_/Si device in the LRS are about 2 and 3 for the 1/2 read scheme and the 1/3 read scheme, respectively. This indicates that the LRS follows Ohmic conduction with a slope of 2. The nonlinearity of the Ni/HfO_2_/Si_3_N_4_/SiO_2_/Si device in the LRS is substantially higher due to its nonlinear I–V characteristics.

**Figure 4:**
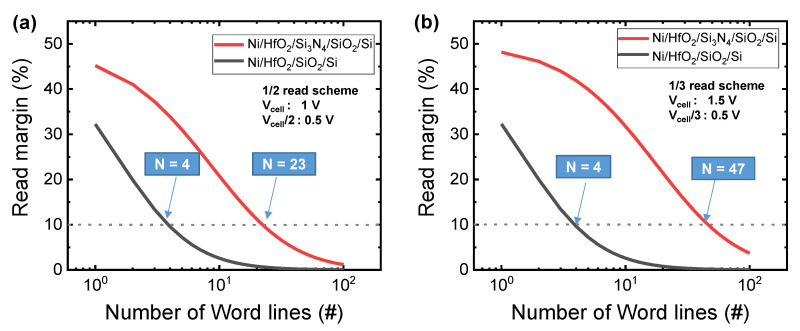
Read margin of Ni/HfO_2_/SiO_2_/Si and Ni/HfO_2_/Si_3_N_4_/SiO_2_/Si devices when applying (**a**) 1/2 read scheme and (**b**) 1/3 read scheme in cross-point array.

Figure 4 shows the read margin as a function of the number of word lines for the Ni/HfO_2_/SiO_2_/Si and Ni/HfO_2_/Si_3_N_4_/SiO_2_/Si devices. Here, the 1/2 read scheme and the 1/3 read scheme are applied to a virtual cross-point array without line resistance. The detailed array read schemes are well known in the literature, and we discuss in detail equation and the scheme in Figure S3. The Ni/HfO_2_/Si_3_N_4_/SiO_2_/Si device shows higher read margin compared to the Ni/HfO_2_/SiO_2_/Si device. This is due to the fact that Ni/HfO_2_/Si_3_N_4_/SiO_2_/Si has higher LRS resistance and nonlinear I–V curves in the LRS. The read margin at the 1/3 read scheme is also higher than that of the 1/2 read scheme.

The conducting path would be formed in the SiO_2_ layer during the set process for the Ni/HfO_2_/Si_3_N_4_/SiO_2_/Si device. The electric field is concentrated within the SiO_2_ layer with consideration of the dielectric constants (HfO_2_: ~20 and SiO_2_: ~4). Therefore, high current cannot be avoided in the LRS after the set process. On the other hand, the overshoot current was mitigated in the Ni/HfO_2_/Si_3_N_4_/SiO_2_/Si device during the set process. This can be explained by the dispersion of the focused electric field of the SiO_2_ layer due to the Si_3_N_4_ layer. The dielectric constant of the Si_3_N_4_ layer (Si_3_N_4_: ~7) is slightly higher than that of SiO_2_ and lower than that of HfO_2_. Therefore, a Si_3_N_4_ layer between the HfO_2_ layer and the SiO_2_ layer is a good buffer layer to reduce the current overshoot.

**Figure 5:**
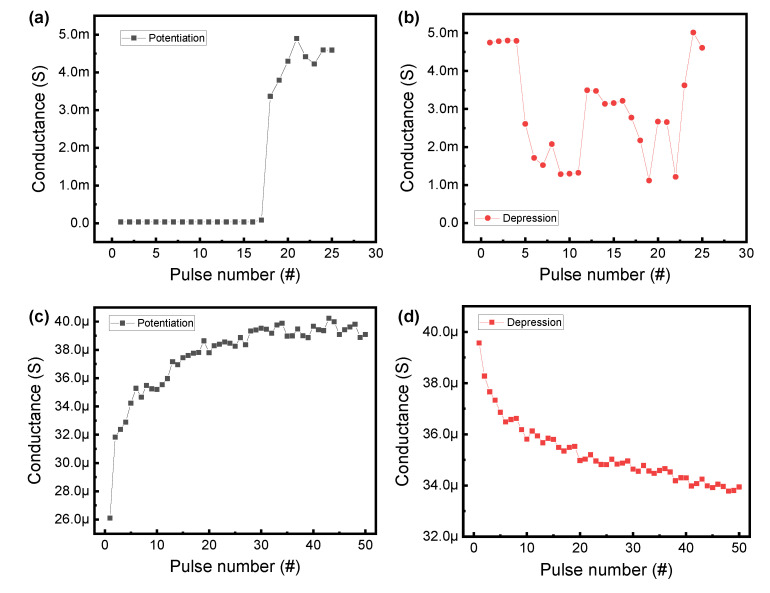
Potentiation and depression characteristics for (**a**,**b**) Ni/HfO_2_/SiO_2_/Si device and (**c**,**d**) Ni/HfO_2_/Si_3_N_4_/SiO_2_/Si device.

Next, we compared the tendency of conductance change as a function of identical pulse during the set and reset process. Figure 5a shows the conductance changes of the Ni/HfO_2_/SiO_2_/Si device for potentiation (set process) and depression (reset process), respectively. The pulse amplitude voltages with a pulse width of 450 µs are 6 V and −3.5 V for potentiation and depression, respectively. The conductance values are extracted from the middle point of read pulse (1 V and 450 µs). For potentiation, the conductance value increases abruptly in response to the 18th pulse. The depression curve shows several fluctuations after the first decrease in conductance. Such randomness and abrupt conductance change are not suitable for a hardware-based neuromorphic synaptic device. On the other hand, the conductance values in the Ni/HfO_2_/Si_3_N_4_/SiO_2_/Si device are gradually controlled by the potentiation and depression pulses (Figure 5b). The voltages of the set pulse and reset pulse are 7 and −4.5 V, respectively, and the pulse width is 450 µs. Further, the read pulse (1 V and 450 µs) is inserted between set pulses or reset pulses to obtain the conductance value. It should be noted that a gradual conductance update is possible when the same pulse is repeatedly applied on the device for potentiation and depression. Moreover, the conductance value of the Ni/HfO_2_/Si_3_N_4_/SiO_2_/Si device is substantially lower than that of the Ni/HfO_2_/SiO_2_/Si device. Therefore, the improved synaptic properties, such as the low energy and multiple conductance, of the Ni/HfO_2_/Si_3_N_4_/SiO_2_/Si device are beneficial for synaptic applications. The conductance update method of the Ni/HfO_2_/Si_3_N_4_/SiO_2_/Si device is suitable for offline learning. To apply it to online learning that provides information by reading conductance values in real time, improvement in variation will be required [33].

## 4. Conclusions

In summary, we fabricated a CMOS-compatible trilayer device (Ni/HfO_2_/Si_3_N_4_/SiO_2_/Si) and characterized its resistive and synaptic characteristics. The TEM and XPS provide the exact dielectric thickness and chemical information of the trilayer device. The Si_3_N_4_ layer could alleviate the concentrated electric field into the SiO_2_ layer in the trilayer design, so the conducting paths are not formed in all dielectrics in the LRS. This property can reduce reset current and provide a nonlinear I–V curve in the LRS. The high nonlinearity in the trilayer device can enlarge the array size in the cross-point array architecture. Finally, we demonstrated that gradual set and reset switching in a trilayer device can be highly suitable for emulating the synaptic behavior of a biological synapse in the human nervous system by controlling multiple conductance.

## Supplementary Materials

The following are available online at https://www.mdpi.com/2079-4991/10/12/2462/s1, Figure S1: The STEM image and EDS line scan of Ni/HfO_2_/Si_3_N_4_/SiO_2_/Si. Figure S2: Definition of nonlinearity of I–V in the LRS. Figure S3: Read operation scheme in virtual cross-point array structure.

## References

[1] Lanza M., Wong H.-S.P., Pop E., Ielmini D., Strukov D., Regan B.C., Larcher L., Villena M.A., Yang J.J., Goux L. (2018). Recommended Methods to Study Resistive Switching Devices. Adv. Electron. Mater..

[2] Linn E., Rosezin R., Kügeler C., Waser R. (2010). Complementary resistive switches for passive nanocrossbar memories. Nat. Mater..

[3] Kim S., Jung S., Kim M.-H., Chen Y.-C., Chang T.-C., Ryoo K.-C., Cho S., Lee J.-H., Park B.-G. (2018). Scaling Effect on Silicon Nitride Memristor with Highly Doped Si Substrate. Small.

[4] Zhou F., Chang Y.F., Fowler B., Byun K., Lee J.C. (2015). Stabilization of multiple resistance levels by current-sweep in SiOx-based resistive switching memory. Appl. Phys. Lett..

[5] Pan F., Gao S., Chen C., Song C., Zeng F. (2014). Recent progress in resistive random access memories: Materials, switching mechanisms, and performance. Mater. Sci. Eng. R Rep..

[6] Maikap S., Banergee W. (2020). In Quest of Nonfilamentary Switching: A Synergistic Approach of Dual Nanostructure Engineering to Improve the Variability and Reliability of Resistive Random-Access-Memory Devices. Adv. Electron. Mater..

[7] Mikhaylov A., Pimashkin A., Pigareva Y., Gerasimova S., Gryaznov E., Shchanikov S., Zuev A., Talanov M., Lavrov I., Demin V. (2020). Neurohybrid Memristive CMOS-Integrated Systems for Biosensors and Neuroprosthetics. Front. Mol. Neurosci..

[8] Kuzum D., Yu S., Wong H.-S.P. (2013). Synaptic electronics: Materials, devices and applications. Nanotechnology.

[9] Graves C.E., Li C., Sheng X., Miller D., Ignowski J., Kiyama L., Strachan J.P. (2020). In-Memory Computing with Memristor Content Addressable Memories for Pattern Matching. Adv. Mater..

[10] Xia Q., Yang J.J. (2019). Memristive crossbar arrays for brain-inspired computing. Nat. Mater..

[11] Ryu H., Kim S. (2020). Pseudo-Interface Switching of a Two-Terminal TaO_x_/HfO_2_ Synaptic Device for Neuromorphic Applications. Nanomaterials.

[12] Choi J., Kim S. (2020). Nonlinear Characteristics of Complementary Resistive Switching in HfAlO_x_-Based Memristor for High-Density Cross-Point Array Structure. Coatings.

[13] Ryu H., Kim S. (2020). Voltage Amplitude-Controlled Synaptic Plasticity from Complementary Resistive Switching in Alloying HfO_x_ with AlO_x_-Based RRAM. Metals.

[14] Cho S., Kim S. (2020). Emulation of Biological Synapse Characteristics from Cu/AlN/TiN Conductive Bridge Random Access Memory. Nanomaterials.

[15] Kim S., Kim H., Jung S., Kim M.H., Lee S., Cho S., Park B.G. (2016). Tuning resistive switching parameters in Si_3_N_4_-based RRAM for three-dimensional vertical resistive memory applications. J. Alloy. Compd..

[16] Emelyanov A.V., Nikiruy K.E., Serenko A.V., Sitnikov A.V., Presnyakov M.Y., Rybka R.B., Sboev A.G., Rylkov V.V., Kashkarov P.K., Kovalchuk M.V. (2020). Self-adaptive STDP-based learning of a spiking neuron with nanocomposite memristive weights. Nanotechnology.

[17] Li D., Wu B., Zhu X., Wang J., Ryu B., Lu W.D., Liang X. (2018). MoS_2_ Memristors Exhibiting Variable Switching Characteristics toward Biorealistic Synaptic Emulation. ACS Nano.

[18] Chand U., Alawein M., Fariborzi H. (2017). Enhancement of Endurance in HfO_2_-Based CBRAM Device by Introduction of a TaN diffusion Blocking Layer. ECS Trans..

[19] Chand U., Huang K.C., Huang C.Y., Ho C.H., Lin C.H., Tseng T.Y. (2015). Investigation of thermal stability and reliability of HfO_2_ based resistive random access memory devices with cross-bar structure. J. Appl. Phys..

[20] Mahata C., Lee C., An Y., Kim M.H., Bang S., Kim C.S., Ryu J.H., Kim S., Kim H., Park B.G. (2020). Resistive switching and synaptic behaviors of an HfO_2_/Al_2_O_3_ stack on ITO for neuromorphic systems. J. Alloy. Compd..

[21] Mikhaylov A., Belov A., Korolev D., Antonov I., Kotomina V., Kotina A., Gryaznov E., Sharapov A., Koryazhkina M., Kryukov R. (2020). Multilayer Metal-Oxide Memristive Device with Stabilized Resistive Switching. Adv. Mater. Technol..

[22] Kim S., Park B.G. (2016). Nonlinear and multilevel resistive switching memory in Ni/Si_3_N_4_/Al_2_O_3_/TiN structures. Appl. Phys. Lett..

[23] Kim M.H., Kim S., Bang S., Kim T.H., Lee D.K., Cho S., Park B.G. (2018). Uniformity Improvement of SiN_x_-Based Resistive Switching Memory by Suppressed Internal Overshoot Current. IEEE Trans. Nanotechnol..

[24] Jung R. (2009). Fermi-Level Pinning at the Poly-Si/HfO_2_ Interface. J. Korean Phys. Soc..

[25] Bommali R.K., Singh S.P., Prakash G.V., Ghosh S., Srivastava P. (2013). Growth and tailoring of physical properties of Si quantum dots in a-SiN_x_:H matrix. Energy Procedia.

[26] Kim T., Koka S., Surthi S., Zhuang K. (2013). Direct Impact of Chemical Bonding of Oxynitride on Boron Penetration and Electrical Oxide Hardening for Nanoscale Flash Memory. IEEE Electron. Dev. Lett..

[27] Ielemini D. (2011). Modeling the Universal Set/Reset Characteristics of Bipolar RRAM by Field- and Temperature-Driven Filament Growth. IEEE Trans. Electron Devices.

[28] Aldana S., García-Fernández P., Rodríguez-Fernández A., Romero-Zaliz R., González M.B., Jiménez-Molinos F., Campabadal F., Gómez-Campos F., Roldán J.B. (2017). A 3D kinetic Monte Carlo simulation study of resistive switching processes in Ni/HfO_2_/Si-n^+^-based RRAMs. J. Phys. D Appl. Phys..

[29] Vandelli L., Padovani A., Larcher L., Bersuker G. (2013). Microscopic Modeling of Electrical Stress-Induced Breakdown in Poly-Crystalline Hafnium Oxide Dielectrics. IEEE Trans. Electron Devices.

[30] Guy J., Molas G., Blaise P., Bernard M., Roule A., Carval G.L., Delaye V., Toffoli A., Ghibaudo G., Clermidy F. (2015). Investigation of Forming, SET, and Data Retention of Conductive-Bridge Random-Access Memory for Stack Optimization. IEEE Trans. Electron Devices.

[31] Roldán J.B., Alonso F.J., Aguilera A.M., Maldonado D., Lanza M. (2019). Time series statistical analysis: A powerful tool to evaluate the variability of resistive switching memories. J. Appl. Phys..

[32] Miranda E., Mehonic A., Ng W.H., Kenyon A.J. (2019). Simulation of Cycle-to-Cycle Instabilities in SiO_x_-Based ReRAM Devices Using a Self-Correlated Process With Long-Term Variation. IEEE Electron Device Lett..

[33] Kim C.-H., Lim S., Woo S.Y., Kang W.M., Seo Y.-T., Lee S.T., Lee S., Kwon D., Oh S., Noh Y. (2018). Emerging memory technologies for neuromorphic computing. Nanotechnology.

