# Supplementary material for: Improved Pulse-Controlled Conductance Adjustment in Trilayer Resistors by Suppressing Current Overshoot
Source: Nanomaterials (Basel). 2020 Dec 9;10(12):2462. doi: 10.3390/nano10122462 (PMC7763874; doi:10.3390/nano10122462)
Supplement: Supplementary file 1 [file nanomaterials-10-02462-s001.pdf]

Supplementary Materials

Improved Pulse-Controlled Conductance Adjustment in Trilayer Resistors by Suppressing Current Overshoot

Hojeong Ryu and Sungjun Kim *

Division of Electronics and Electrical Engineering, Dongguk University, Seoul 04620, Korea; hojeong.ryu95@gmail.com
* Correspondence: sungjun@dongguk.edu

(a)

(b)

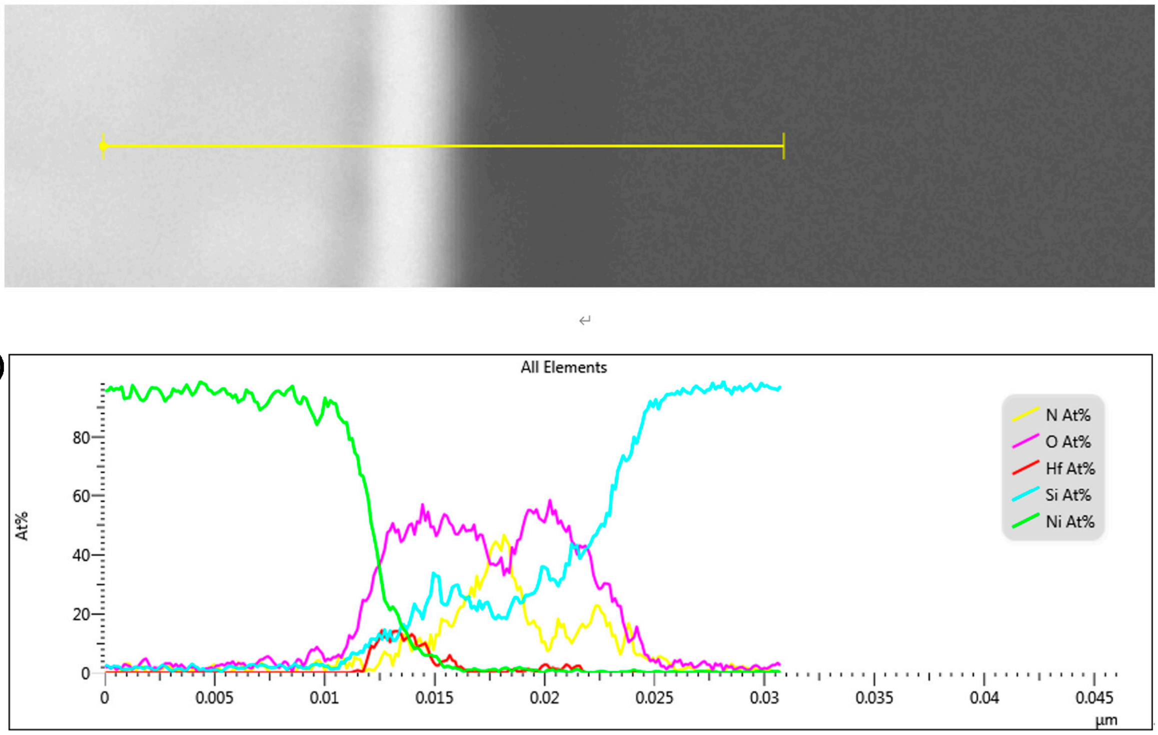

Figure S1. (**a**) STEM image and (**b**) EDS line scan of Ni/HfO_2/Si_3N_4/SiO_2/Si device.

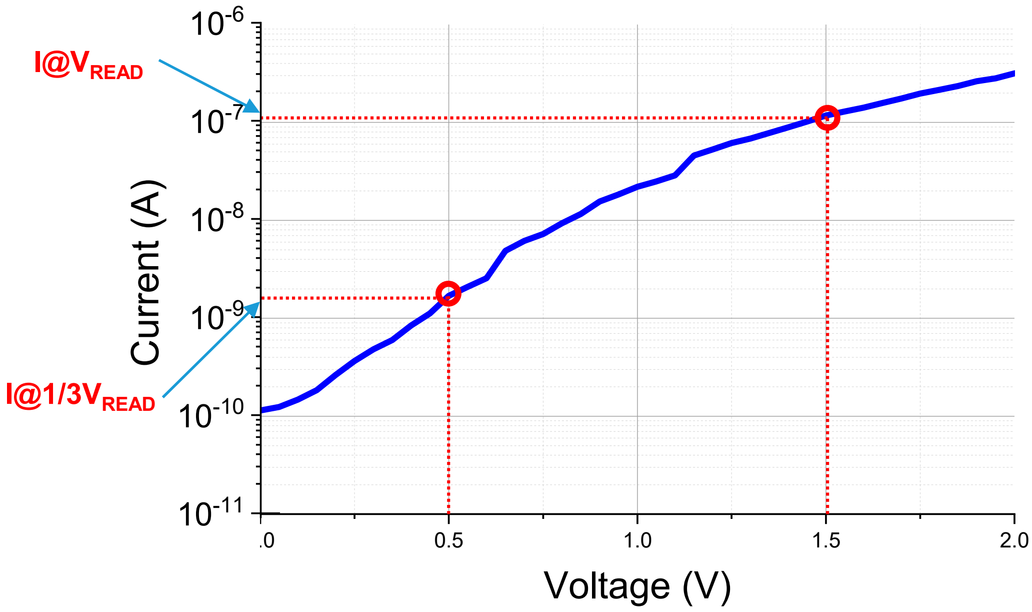

Figure S2. Definition of nonlinearity of I–V in the LRS.

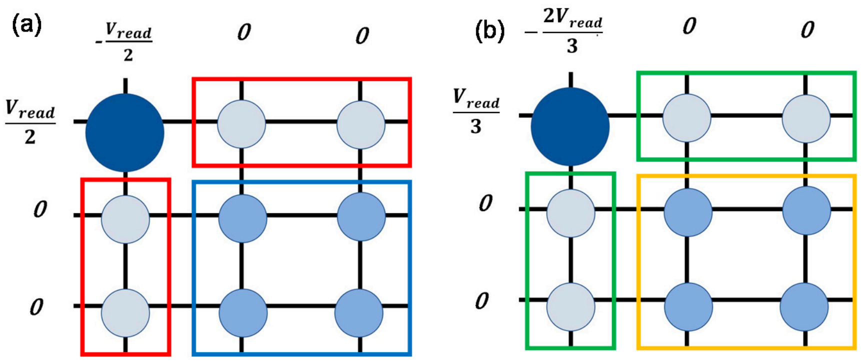

½ scheme

$$\text{Read margin} = \left(\frac{R_{pu}}{\left(R_{LRS}(V_{cell})||(\frac{2R_{LRS}(\frac{V_{cell}}{2})}{N-1}+\frac{R_{LRS}(Vmi_{n})}{(N-1)^2})\right)+Rpu} - \frac{R_{pu}}{\left(R_{HRS}(V_{cell})||(\frac{2RLR_{S}(\frac{V_{cell}}{2})}{N-1}+\frac{R_{LRS}(Vmi_{n})}{(N-1)^2})\right)+Rpu}\right) \times 100\ (\%)$$

1/3 scheme

$$\text{Read margin} = \left(\frac{R_{pu}}{\left(R_{LRS}(V_{cell})||(\frac{R_{LRS}(\frac{V_{cell}}{3})}{N-1}+\frac{R_{LRS}(\frac{2V_{cell}}{3})}{N-1}+\frac{R_{LRS}(Vmi_{n})}{(N-1)^2})\right)+Rpu} - \frac{R_{pu}}{\left(R_{HRS}(V_{cell})||(\frac{R_{LRS}(\frac{V_{cell}}{3})}{N-1}+\frac{R_{LRS}(\frac{2V_{cell}}{3})}{N-1}+\frac{R_{LRS}(Vmi_{n})}{(N-1)^2})\right)+Rpu}\right) \times 100\ (\%)$$

Figure S3. Read scheme: (**a**) half bias scheme and (**b**) 1/3 bias scheme and their equation for read margin.
